# Sero-prevalence of syphilis and associated risk factors among pregnant women attending antenatal care at an urban-poor health centre in Kampala, Uganda: a cross-sectional study

**DOI:** 10.11604/pamj.2024.47.129.31622

**Published:** 2024-03-22

**Authors:** Andrew Simiyu, Collins Grace Kalanga Atuheire, Martha Taremwa, Sarah Nabwire Ssali, Frank Norbert Mwiine, Clovice Kankya, Kizito Kahooza Mugimba

**Affiliations:** 1 Department of Biosecurity, Ecosystems and Veterinary Public Health (School of Biosecurity, Biotechnology and Laboratory Sciences (SBLS)), College of Veterinary Medicine, Animal Resources and Biosecurity, Makerere University, Kampala, Uganda,; 2 School of Women and Gender Studies, College of Humanities, Makerere University, Kampala, Uganda,; 3 Department of Biomolecular and Biolaboratory Sciences (School of Biosecurity, Biotechnology and Laboratory Sciences (SBLS)), College of Veterinary Medicine, Animal Resources and Biosecurity, Makerere University, Kampala, Uganda

**Keywords:** Syphilis, HIV, pregnant women, risk factors

## Abstract

**Introduction:**

syphilis and its outcomes remain a healthcare system burden with adverse consequences such as stillbirths, neonatal deaths and spontaneous abortions among others. The situation might have worsened because the COVID-19 pandemic has caused a major attention drift from other diseases. Additionally, much as testing for syphilis is a routine practice among pregnant mothers, its proportion is not known in urban health care setting. A study to determine the prevalence of syphilis among pregnant mothers in an urban poor setting is warranted.

**Methods:**

a cross-sectional study was conducted among pregnant women who attended antenatal care at Kawaala Health Centre IV in Kampala Capital City between December 2019 to March 2020. Informed consent was sought from study participants prior to data collection using structured questionnaires. Whole blood was collected and tested using SD Bioline HIV/syphilis duo rapid test kit (SD Standard Diagnostics, INC, Korea). Data analysis was done using STATA 14.2.

**Results:**

one thousand one hundred and sixty-nine pregnant women participated in the study, with a mean age of 25 years. About 27% of them had completed only primary-level education. Approximately 6% of the participants were HIV seropositive. The prevalence of syphilis was 5.9% (69/1169). HIV positivity (aOR: 4.13, 95%CI: 2.05-8.34), elevated blood pressure (aOR: 2.84, 95%CI: 1.42-5.69), and status of previous pregnancy (aOR: 0.21, 95%CI: 0.05-0.89) were significant predictors of the risk of syphilis among pregnant women in this setting.

**Conclusion:**

the prevalence of syphilis among pregnant women in urban poor settings is not low and so must not be underestimated. The potential drivers of syphilis among pregnant women are HIV, elevated blood pressure, and status of previous pregnancy. There should be increased awareness about routine syphilis testing among pregnant mothers attending antenatal care.

## Introduction

Syphilis caused by *Treponema pallidum* subspecies *pallidum* remains endemic in the world accounting for an estimated six million infections annually [1]. The disease is more pronounced in low-income countries with high infection rates that vary from region to region [2]. The World Health Organization set an ambitious plan to reduce syphilis by 90% by 2030 [3]. This target should be supported by regular epidemiological surveillance in high-burden areas to guide intervention by Newman *et al*. [1]. Particularly, the effect of syphilis in pregnant women resulting in fetal death, and vertical and perinatal transmission should be of concern [4]. It is estimated that close to 300,000 fetal deaths are associated with syphilis infection globally [4]. This in addition to other indirect effects on fetal development that could impact child development, estimates put 100 neonatal syphilis cases per 100,000 births in low- and middle-income countries (LMICs) [5]. In sub-Saharan Africa alone, close to one million pregnant women could be infected with syphilis. Despite the putative low antenatal screening in East Africa, 2.7% (95% CI: 2.06-2.29) prevalence has been reported in pregnant women [6], underscoring the need to scale up syphilis screening. It is therefore important that pregnant women are screened regularly to control the effects of syphilis.

Syphilis and Human Immuno-deficiency Virus (HIV) are mainly transmitted through risky sexual behavior in both heterosexuals and men having sex with men (MSM). During the early stages of HIV, the prevalence of other Sexually Transmitted Infections (STIs) including syphilis had reduced save for developing countries mainly due to safe sex campaigns, only to register an increase in the early part of the 21^st^ century [7]. There is an intricate relationship between HIV and syphilis. The ulcers associated with clinical syphilis increase the risk of HIV infection [8], whereas the progression of HIV into Acquired Immuno-deficiency Syndrome (AIDS) has a significant outcome on the prognosis of syphilis management resulting mainly from immunosuppression [9]. In a study in Mwanza, Tanzania, management of STIs in a study population resulted in a 38% reduction in HIV infection rate illuminating the possible co-morbidity effects [10]. However, in a related study conducted in Rakai in Uganda, no corresponding reduction was recorded in HIV community infection signaling differences in the response [11]. Regular HIV screening is mandatory for all women attending antenatal care in Uganda. This has aided significant progress in the control of mother-to-child transmission and the general HIV burden in the population. Despite recommendations by the Ministry of Health on regular screening of syphilis in women attending antenatal clinics, this has not yet been fully implemented. Early detection and treatment of syphilis in pregnant women is important and cost-effective in reducing the impact of the disease [12]. It is important that we understand the interaction of these two important Sexually Transmitted Infection (STI) to plan effective control strategies in pregnant women. Following infection, antibodies are produced as early as two weeks and could last up to ten years depending on the isotype and disease progression/management [13]. *Treponema pallidum* elicits two antibodies that have become important in the diagnosis of syphilis. The first is called reagin or nontreponemal antibody. It is a nonspecific antibody that appears several weeks after infection; its recognition is the basis of screening tests for HIV/syphilis duo kit. Most syphilis cases are diagnosed serologically using tests that detect antibodies directed at either lipid (non-treponemal test) or specific treponemal antigen (treponemal test) [14].

Treatment of syphilis relies on use of antimicrobial agents like benzathine benzylpenicillin. However, some cases of treatment failure were reported in a study on immigrant workers in Israel signaling impending difficulty in clinical management of syphilis [15]. The use of other agents like macrolides is hampered by drug resistance that has been reported, signaling the need to scale up control strategies that reduce the burden of infection. Untreated syphilis in pregnant women is a major cause of morbidity and mortality, resulting in spontaneous abortion, hydrops, intrauterine growth restriction, malformations, fetal death and stillbirths, preterm or low birth weight infants, neonatal death, and syphilis infection in infants. In addition, maternal syphilis leads to an increase of mother-to-child transmission (MTCT) of HIV [16]. There is a paucity of data concerning the burden of syphilis among antenatal mothers in Uganda. In this study, we investigated the seroprevalence of syphilis in pregnant women attending antenatal care in Kawaala Health Center IV and the associated risk factors.

## Methods

**Study design:** this was a cross-sectional study that was conducted among pregnant women who attended antenatal care at Kawaala Health Centre IV in Kampala Capital City Authority (KCCA). We have been able to report our findings in accordance with STROBE guidelines [17].

**Study setting:** the study was conducted at Kawaala HCIV in Kampala Capital City Authority (KCCA) between the months of December 2019 and March 2020 inclusive. Kawaala HCIV is part of the national healthcare system of Uganda and offers secondary and tertiary healthcare including maternal and child health care. Kawaala HC IV is located in Kawaala, a Kampala suburb found in Lubaga Division in Kampala City Authority. It is bordered by Nabweru to the North, Kazo to the Northeast, Makerere to the East, Naakulabye to the South, Kasubi to the Southwest, and Namungoona to the West. This is approximately 5 kilometres (3.1 mi), by road, north of Kampala's central business district. The coordinates of Kawaala HC IV are 0.3408, 0.3408. Kawaala is a peaceful residential neighborhood, with an occasional middle and high-class residence. It offers services such as outpatient care. Antenatal care, maternity services, post-natal care (MCH), Prevention of Mother to Child Transmissions (PMTCT) care, HIV/AIDS care and counselling, laboratory services, outreaches, and immunization among others.

**Sample size:** the sample size was statistically calculated based on single population proportion by Sullivan L [18], by taking the 17% prevalence of syphilis infection among pregnant women in Uganda [16] and a standard normal deviation of 1.96 for a confidence limit of 95%, where:


$$n=\frac{{\left(z\beta +z\alpha \right)}^{2}p\left(1-p\right)}{d^{2}}$$

Where n = the desired sample size; Z_α_= the standard normal deviation at 5%=1.96; Z_β_=one left-tailed z statistic at the area of 20% (80% statistical power) = 0.84; p = proportion of pregnant women with syphilis infection attending antenatal care; the prevalence of syphilis among pregnant women attending antenatal care in Uganda is 17% [16]. However, this reference of 2011 was the only available national data during the conduct of this study. d = the permissible error at 0.05, and catering for design effect; adjusted sample size would a non-response of 10% (r=0.1). The final sample size was 985.

**Sampling strategies:** only one health center was selected in Kampala Capital City Authority and this was Kawaala Health Center IV (HCIV) which provides antenatal care services. Consecutive enrollment of mothers sampling was used to recruit the study participants. All mothers who came to attend ANC met the eligibility criteria, and consented to participate in the study were sampled until the minimum required sample size of at least 985 was obtained and more participants recruited up to 1169. This enabled us to get a favorable sample size for this study. The eligibility criteria were such that all pregnant mothers from Uganda and who have not had recent travel or obtained antenatal care from outside Uganda for the current pregnancy and who consented to participate in the study. Those who were on current syphilis treatment (1.45% (17/1169)) were excluded. We based on self-report by the mother without laboratory confirmation and this could have introduced missed opportunity for syphilis testing and hence introduced some level of measurement and information bias. The non-response rate was 2.48% (29/1169).

**Data collection methods and tools:** data was collected by trained research nurses and laboratory technologists using a structured questionnaire tool designed in three sections (Annex 1). The first section was sociodemographic data, the second one was geographic and obstetric variables, and the third involved laboratory findings to capture HIV and syphilis statuses. The questionnaire was designed in English, however, it was translated in the local language, “Luganda” during data collection for mothers who were never fully conversant with English. Regarding the syphilis infection status, whole blood was collected from the pregnant women (attending Kawaala Hospital Antenatic Clinic) using the finger prick/lancet methods and tested using the SD BIOLINE HIV/syphilis duo rapid test, (manufacturer; SD Standard Diagnostic, INC. Korea). The duo test kit runs HIV and syphilis testing simultaneously. Blood collection and testing were carried out by a trained laboratory technologist (Mr. Simiyu Andrew) assisted by nurses. Laboratory testing took place at Kawaala Hospital Laboratory.

**Data quality control:** the questionnaire design was carried out by the three project team members and in consultation with senior laboratory professionals in syphilis surveillance. Questionnaire pretesting was done and checked and for validity and reliability. The quality of the laboratory results was ensured by using valid syphilis/HIV duo test kits, using competent laboratory personnel and the testing was carried out in strict accordance with the manufacturer´s instruction (Annex 1).

### Variables

**Socio-demographic data:** age, weight, height, blood pressure, marital status, stage of pregnancy, education, employment status, pregnancy history, and ethnicity were collected using a structured questionnaire whereas the laboratory results were obtained from blood samples collected from study participants and tested at the ANC point of care testing for both syphilis and HIV. The outcome variable was syphilis infection measured as a binary variable (0=negative; 1=positive).

**Data analysis:** summary statistics for age, body mass index (BMI) measured as weight over square meters, and systolic blood pressure (SBP) were medians and interquartile ranges since they were skewed whereas the number of sexual partners was summarized as mean and standard deviation (SD). Categorical variables such as marital status, geopolitical divisions of Kampala, education level, employment status, previous pregnancy status, HIV status, ethnic group, and trimester were summarized as frequencies, n, and percentages, % as presented in Table 1.

**Table 1:** socio-demographic and clinical characteristics of pregnant women attending antenatal care (ANC) at Kawaala Health Centre IV, Kampala, Uganda

Variable	Summary measure
Median age in years (IQR)	25(23-29)
Median BMI in kgm^2^ (IQR)	25.07(22.84-28.13)
Median systolic blood pressure in mmHg (IQR)	110(105-120)
**Marital status n (%)**	
Married	1130(96.7)
Separated	39(3.3)
Mean number of sexual partners (sd)	1.02(0.17)
**Geo-political divisions of Kampala, n (%)**	
Lubaga	655(56.03)
Nakawa	6(0.51)
Kawempe	331(28.31)
Kampala central	16(1.37)
Makindye	2(0.17)
Wakiso	151(12.92)
Other	8(0.68)
**Education level, n (%)**	
Primary	312(26.69)
Secondary	715(61.16)
Tertiary	142(12.15)
**Employment status, n (%)**	
None	593(50.73)
informal	457(39.09)
Formal	119(10.18)
**Status of previous pregnancy, n (%)**	
Alive	859(87.74)
Dead	120(12.26)
**HIV status, n (%)**	
Negative	10954(93.58)
Positive	75(6.42)
**Ugandan ethnic groups, n (%)**	
Bantu	1075(91.96)
Nilo-Hamites	33(2.82)
Nilotics	26(2.22)
Adhola	18(1.54)
Luo	8(0.68)
Sabiny	9(0.77)
**Trimester n (%)**	
First	139(11.89)
Second	702(60.05)
Third	328(28.06)

IQR: interquartile range; BMI: body mass index

The prevalence was analyzed as frequencies and percentages, % of positive both overall and in various strata of the geopolitical division of Kampala and ethnic groups. Ninety-five percent confidence intervals were constructed for each category to ascertain any statistical significance as shown in Table 2.

**Table 2:** prevalence of syphilis among pregnant women attending antenatal care (ANC) at Kawaala Health Centre IV in Kampala, Uganda

Variable	Total number of cases	%	95% confidence interval
Syphilis (overall)	69	5.9	4.7-7.4
**Syphilis by geo-political divisions of Kampala**			
Lubaga	39	56.52	44.4-67.9
Nakawa	0	0.00	
Kawempe	23	33.33	23.0-45.5
Kampala central	1	1.45	1.94-10.0
Makindye	0	0.00	
Wakiso	6	8.70	3.88-18.35
Other	0	0.00	
**Syphilis by Ugandan ethnic groups**			
Bantu	60	86.96	76.47-93.19
Nilo-Hamites	3	4.35	11.37-12.96
Nilotics	3	4.35	11.37-12.96
Adhola	1	1.45	0.19-10.02
Luo	2	2.90	0.7-11.2
Sabiny	0	0.00	

We performed univariable (using frequencies, and percentages), bivariable (using logistic regression), and multivariable analysis (using logistic regression). Probability values (P-value) are obtained from z-statistics computed during logistic regression. At bivariable logistic regression, we obtained crude odds ratios (cORs) whereas multivariable logistic regression provided adjusted Odds Ratios (aORs). Variables, to qualify for multivariable analysis, were selected based on a P-value cut-off of 0.25 at the bivariable analysis stage. In Table 3, BMI was categorized as underweight (<18.5 kg/m^2^), normal (18.5 to <25 kg/m^2^), overweight (25 to <30 kg/m^2^), obese (30 kg/m^2^ and above) while systolic blood pressure was categorized as normal (<120 mmHg), elevated (121-129 mmHg), and hypertensive (130 mmHg and above). At multivariable analysis, interaction or effect modification was carried out using a likelihood ratio test was carried out followed by assessing for confounding. For the latter, we used a confounding proportion cut-off of 10%. At multivariable analysis, statistical significance was considered at p≤0.05 and a 95%CI not crossing the null, Ho: OR=1. We also performed a Spearman correlation between syphilis and HIV positivity (Table 4). All statistical analyses were performed in Stata 14.2 (StataCorp, Lakeway Drive, USA Texas).

**Table 3:** binary logistic regression of syphilis on socio-demographic and clinical characteristics of pregnant women attending antenatal care (ANC) at Kawaala Health Centre IV in Kampala, Uganda

	Univariate analysis	Bivariate analysis	Adjusted analysis
Variable	Syphilis n (%)	cOR(95%CI), p-value	aOR(95%CI), p-value
**Blood pressure in mmHg**			
Normal	48(69.57)	1.00	1.00
Elevated	13(18.84)	2.48(1.30-4.75), 0.006	2.84(1.42-5.69), 0.003
hypertensive	8(11.59)	1.45(0.67-3.16), 0.34	1.49(0.68-3.31), 0.33
**Status of pregnancy**			
Alive	60(96.77)	1.00	1.00
Dead	2(3.23)	0.23(0.05-0.94), 0.04	0.21(0.05-0.89), 0.034
**HIV status**			
Negative	56(81.16)	1.00	1.00
Positive	13(18.84)	3.89(2.02-7.49), <0.001	4.13(2.05-8.34), <0.001
**Ugandan ethnic groups**			
Bantu	60(86.96)	1.00	1.00
Nilo-Hamites	3(4.35)	1.69(0.50-5.70), 0.40	1.47(0.40-5.30), 0.45
Nilotics	3(4.35)	2.21(0.64-7.56), 0.21	2.11(0.44-6.61), 0.25
Adhola	1(1.45)	1.00(0.13-7.60), 1.00	-
Luo	2(2.90)	5.64(1.11-28.53), 0.04	
Sabiny	0(0.0)		

**Table 4:** correlation between HIV-syphilis co-infection and other predictors

Variable^g^	Correlation coefficient (r)	p-value
HIV versus syphilis	0.13	<0.001
**Syphilis-HIV co-infection^f^ versus:**		
Age	0.03	0.26
BMI	-0.006	0.84
SBP	0.04	0.15
Employment	0.08	0.009
Number of sexual partners	-0.01	0.72
Ethnicity	-0.027	0.36

f prevalence of syphilis-HIV co-infection: 1.1%, (13/1,169; [95%CI 0.6%-1.9%]); ^g^the other factors were non-significantly correlated; BMI: body mass index; SBP: systolic blood pressure

**Ethical considerations:** the study was conducted after obtaining clearance from the Research Ethical Committee of Kampala Capital City Authority under the Directorate of Public Health and Environment with reference number DPHE/KCCA/201/17.

## Results

**Figure 1:**
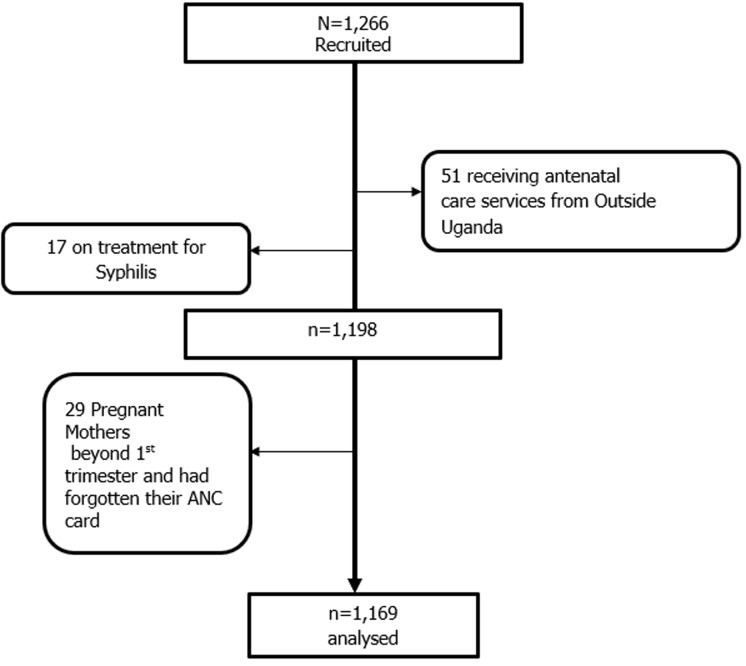
study flow diagram

**Study flow diagram:** as shown in Figure 1, 1,266 participants were enrolled, and due to various conditions, 1,169 participants were analyzed.

**Socio-demographic and clinical characteristics of pregnant women attending ANC at Kawaala Health Centre IV Kampala, Uganda:** in this study, the total number of enrolled participants was 1,169 and their median age was 25 years, median BMI of 25.07 kgm^2^, and median systolic blood pressure of 110 mmHg. Ninety-seven percent of the participants were married and the majority of them came from Lubaga Division, followed by the Kawempe Division. About 61% of the participants had attained secondary education, 26% with primary education, and 12% with tertiary education. The majority of the pregnant women were in their second trimester, about 39% of them were employed in the informal sector whereas only 10% were formally employed. About 88% of the pregnant women had previously given birth to a live baby. Six percent of the participants were HIV-positive. The majority of the women were of Bantu ethnicity while the Sabiny ethnic group was the smallest (Table 1).

**Prevalence of syphilis among pregnant women attending ANC at Kawaala Health Centre IV in Kampala, Uganda:** the overall prevalence of syphilis in this study was 5.9% with the majority of the syphilis cases coming from Lubaga Division, followed by Kawempe with no syphilis cases being registered from the Nakawa and Makindye Divisions. About 87% of the syphilis cases came from the Bantu-speaking ethnicity. Nilotics and Nilo-Hamites both had about 5% of the syphilis cases, Adhola had 2%, and Luo 3% with no syphilis cases among the Sabinys (Table 2).

**Association between syphilis on socio-demographic and clinical characteristics of pregnant women attending ANC at Kawaala Health Centre IV in Kampala, Uganda:** in this study, pregnant women who had elevated blood pressure were found to be about three times more likely to have syphilis (adjusted odds ratio (aOR): 2.84,95%CI: 1.42-5.69; p=0.003) than those pregnant women who had normal blood pressure. Pregnant women who lost their previous pregnancy were found to be 79% less likely to have syphilis than those pregnant women who gave birth to a live baby previously (aOR: 0.21, 95%CI: 0.05-0.89; p=0.034). HIV-positive women were found to be four times more likely to have syphilis (aOR: 4.13, 95%%CI: 2.05-8.34; p<0.001) compared to their HIV-negative counterparts (Table 3).

**Correlation between HIV-Syphilis co-infection and other predictors:** the prevalence of syphilis-HIV co-infection in this study was 1.1%, (13/1,169; (95%CI 0.6%-1.9%)). HIV-positivity was positively correlated with syphilis infection (p<0.001). The employment status of a pregnant woman was positively correlated with syphilis-HIV co-infection with a formally employed woman being at higher risk of co-infection than a non-formally employed woman (Table 4).

## Discussion

Approximately 6% of the participants were HIV seropositive. The prevalence of syphilis was 5.9% (69/1169). HIV positivity (aOR: 4.13, 95%CI: 2.05-8.34), elevated blood pressure (aOR: 2.84, 95%CI: 1.42-5.69), and status of previous pregnancy (aOR: 0.21, 95%CI: 0.05-0.89) were significant predictors of the risk of syphilis among pregnant women in this setting.

The overall prevalence of syphilis in this study was two times higher than that reported previously in Mayuge District in Uganda [19]. The difference may be attributed to geographical location. Our study area, Kawaala is located in an urban setting where increased sexual activity is more likely. In addition, health-seeking behaviour for urban mothers is quite higher than those in rural settings, a condition that can increase the prevalence of the outcome under investigation. Our findings corroborate those of Niama *et al*. 2017 and Geremew *et al*. 2021, where they found syphilis infection more in town settings of Republic of Congo and Ethiopia than rural settings respectively [20,21]. In a related study of men having sex with men (MSM) in Tanzania, the rate of STI infection was higher in major towns compared to countryside urban areas [22]. In Rwanda, urban dwellers were reported to be twice as likely to test positive for syphilis compared to rural participants in a study [23]. A population-based study in Zambia reported a higher risk for HIV/syphilis co-infection in urban areas than in rural populations [24]. The observed prevalence in this study is higher than that reported as the average for the East African region, 4.6% [25]. However, the prevalence by Joseph Davey DL *et al*. was based on projections at the backdrop of low screening of syphilis in pregnant women and may not be conclusive [25]. In a meta-analysis by Hussen *et al*. 2019, the prevalence of syphilis in East Africa was estimated at 3.6% [6]. This only underscores the fact that regular screening of pregnant women is a step in the right direction towards control of syphilis and should be scaled up.

Several factors have been advanced to contribute to the prevalence of syphilis in pregnant women including but not limited to low economic status, level of education, ethnicity, prostitution, and others [26]. In the current study, we investigated several factors including tribe, level of education, sexual behavior, and history of pregnancy. There was a high prevalence of syphilis in Bantu ethnic grouping, but this could have been skewed by the high representation in the study population and therefore needs to be studied in detail if there are meaningful effects of ethnic grouping on syphilis. Pregnant women who lost their previous pregnancies were found to be 79% less likely to have syphilis infection. This is possibly so as such mothers tend to seek medical advice much more than those mothers without previous loss of pregnancy. This differential sensitization makes them extremely more careful with regular screening and taking STI preventive measures to rule out any risk that could potentially lead to the loss of further baby (ies). This differs significantly from what was reported in Rwanda where syphilis infection was greatly influenced by previous abortion [27]. The reason for this variation could be attributed to the difference in sample size but underscores the importance of epidemiological surveys in keeping pace with the disease dynamics.

We observed in this study that syphilis and HIV were positively correlated, this is attributed to the same source and somewhat mechanism of infection [28]. The associated genital ulcers in both heterosexuals and MSM have been reported to increase the risk of HIV transmission [29]. In turn, the progression of HIV to AIDs dampens the immune system and increases the risk of syphilis infection [30]. Our analysis of the sociodemographic factors indicated that employment status (as one gets from “none”, “informal”, to “formal” status) is significantly correlated with HIV-syphilis co-infection. This is not reported anywhere in similar studies, but we hypothesize that most formally employed women in urban settings are not married, and in this study, only 10% of such mothers were married compared to 52% of non-employed mothers who were married. Therefore, formally employed women are likely to have multiple sexual partners relative to their counterparts who are not formally employed at all. Reports from the USA-CDC projected that syphilis increases up to 5-fold the risk of acquiring HIV and syphilis has potential to increase viral load in already HIV-positive gay men [31]. In a prevalence study in Eastern Ethiopia, the seroprevalence of syphilis was significantly higher in HIV-positive pregnant women than in HIV-negative ones [32]. In a cross-sectional study in Rwanda, a 55.6% increase in the prevalence of syphilis was observed in HIV-positive participants compared with a 21.6% reduction in HIV-negative participants [33]. A study by Mukanyangezi *et al*. in 2018 [33] involving 237 HIV-positive women from two university hospitals in Rwanda reported a syphilis prevalence of 10%, the highest reported in the Great Lakes´ Region. Research has indicated that the interaction between HIV and syphilis during infection has influence on disease progression to neurosyphilis and treatment failure [34]. With fluctuating trends of HIV infection, it becomes more imperative to conduct regular research on the interaction of these pathogens to guide control [35].

Elevated blood pressure was associated with an increased risk of syphilis, and this is possibly so due to complex immune suppression associated with hypertension [36].

**Limitations:** we cannot ascertain temporal relationship between HIV and syphilis since this was a cross-sectional study. This is potential of non-differential bias i.e toward the null. There is therefore a possibility of diluting the effect that we obtained. Additionally, this quantitative study has less generalizability because of the selection of participants from a health facility setting. Our sample size computation was based on the estimated prevalence of 17% which was obtained 9 years apart, however, being a national database, 17% was assumed to be a representative estimate for prevalence among antenatal mothers. Finally, since we did not carry out random sampling, the study results might have suffered selection bias.

**Recommendation:** follow-up for syphilis routine screening among HIV-positive antenatal mothers should be highly emphasized. High blood pressure should be indicated as a red zone for possible STIs. Future studies are necessary to cover a large number of health facilities blending both rural and urban health care centers.

## Conclusion

Syphilis among pregnant mothers is not given the required attention in urban poor settings of many developing countries especially those that are HIV seropositive. The prevalence of syphilis among pregnant women who attended ANC from Kawaala Health Centre IV, Kampala Uganda between December 2019 and March 2020 was 5.9%. Syphilis-positive pregnant women who were co-infected with HIV were four times more likely to have syphilis infection than their HIV-negative counterparts. Pregnant women with elevated blood pressure were more likely to have syphilis than their counterparts with normal blood pressure. This study also found that pregnant women who lost their previous pregnancy had fewer chances of having syphilis as compared to those women who had a successful pregnancy outcome.

### What is known about this topic

- *The syphilis burden in Uganda is already known among pregnant women and it staggers around 4% as of 2008;*
- *It is known that HIV positivity is positively associated with syphilis infection*.

### What this study adds

- *This is the first study to report the sero-prevalence of syphilis in an urban poor community during the COVID-19 lockdown period where access to health care was a big challenge;*
- *Our study has shown that antenatal mothers with elevated blood pressure have a higher risk of syphilis after controlling for other factors*.

## Annex

**Annex 1**: questionnaire on sociodemographic data, geographic and obstetric variables, and laboratory findings to capture HIV and syphilis statuses (PDF-84 KB)
